# MRI Radiomics in Prostate Cancer: A Reliability Study

**DOI:** 10.3389/fonc.2021.805137

**Published:** 2021-12-21

**Authors:** Fabrizio Urraro, Valerio Nardone, Alfonso Reginelli, Carlo Varelli, Antonio Angrisani, Vittorio Patanè, Luca D’Ambrosio, Pietro Roccatagliata, Gaetano Maria Russo, Luigi Gallo, Marco De Chiara, Lucia Altucci, Salvatore Cappabianca

**Affiliations:** ^1^ Department of Precision Medicine, University of Campania Luigi Vanvitelli, Naples, Italy; ^2^ Istituto Diagnostico Varelli, Naples, Italy

**Keywords:** prostate cancer, radiomics, texture, target therapy, magnetic resonance imaging (MRI)

## Abstract

**Background:**

Radiomics can provide quantitative features from medical imaging that can be correlated to clinical endpoints. The challenges relevant to robustness of radiomics features have been analyzed by many researchers, as it seems to be influenced by acquisition and reconstruction protocols, as well as by the segmentation of the region of interest (ROI). Prostate cancer (PCa) represents a difficult playground for this technique, due to discrepancies in the identification of the cancer lesion and the heterogeneity of the acquisition protocols. The aim of this study was to investigate the reliability of radiomics in PCa magnetic resonance imaging (MRI).

**Methods:**

A homogeneous cohort of patients with a PSA rise that underwent multiparametric MRI imaging of the prostate before biopsy was tested in this study. All the patients were acquired with the same MRI scanner, with a standardized protocol. The identification and the contouring of the region of interest (ROI) of an MRI suspicious cancer lesion were done by two radiologists with great experience in prostate cancer (>10 years). After the segmentation, the texture features were extracted with LIFEx. Texture features were then tested with intraclass coefficient correlation (ICC) analysis to analyze the reliability of the segmentation.

**Results:**

Forty-four consecutive patients were included in the present analysis. In 26 patients (59.1%), the prostate biopsy confirmed the presence of prostate cancer, which was scored as Gleason 6 in 6 patients (13.6%), Gleason 3 + 4 in 8 patients (18.2%), and Gleason 4 + 3 in 12 patients (27.3%). The reliability analysis conversely showed poor reliability in the majority of the MRI acquisition (61% in T2, 89% in DWI50, 44% in DWI400, and 83% in DWI1,500), with ADC acquisition only showing better reliability (poor reliability in only 33% of the texture features).

**Conclusions:**

The low ratio of reliability in a monoinstitutional homogeneous cohort represents a significant alarm bell for the application of MRI radiomics in the field of prostate cancer. More work is needed in a clinical setting to further study the potential of MRI radiomics in prostate cancer.

## Background

Prostate cancer (PCa) is the most frequent male malignancy with 1.4 million new diagnoses per year worldwide (1), and it represents the sixth leading cause of cancer death in men (2).

A pivotal role in clinical workup for PCa patients is played by pathology through prostate biopsy (3, 4) and the Gleason score assessment. The importance of tissue examination was recently proven when, among the other prognostic factors, tumor heterogeneity defined through genomic analyses showed to directly impact on overall survival and cancer control (5–8). Today, through biopsy and molecular assays, we can assess lesion molecular pattern that is more and more important given that PCa may show through several clinical scenarios. In fact, the clinical presentation of prostate cancer can range from a slow, localized, and indolent disease to a rapidly evolving lethal metastatic disease (9).

Adopting radiomics as an available and cost-effective tool, longitudinal monitoring as well as whole tumor examination is possible (e.g., to assist diagnostic random biopsies when sampling errors are likely to occur, due to intratumoral heterogeneity). The widespread use of medical imaging and the increasingly unleashed potential of radiomic features (RF) have made non-invasive examination as important as the invasive one (which still remains the gold standard to get a proper diagnosis of malignancy). Given its current role, radiomics, as a quantitative data extraction method, has received high expectations to become the new frontier of precision medicine and clinical imaging. With large image datasets and a “population-imaging” approach, RF may also be used to discover previously unknown markers and pattern of disease evolution, progression, and treatment response (10–13). Because of the discrepancies in identifying cancer lesions radiographically and within the various acquisition protocols, PCa represents a difficult playground for radiomics although RF could be truly helpful in many fields. For instance, radiomics could be applied for tumor localization and detection, as well as for prediction of prognosis, esteemed for a successful treatment (14, 15), or it could be used with follow-up imaging and combined with preintervention data to stratify the risk of patients for a personalized medicine approach.

The aim of this study was to investigate the reliability of radiomics, focusing on the robustness of radiomic features, in prostate cancer detection.

## Methods

### Population

For the present study, we retrospectively evaluated a homogeneous cohort of consecutive patients with a PSA rise that underwent multiparametric MRI imaging of the prostate before biopsy in a defined time period from July 2017 to March 2019. All the patients subsequently underwent a random prostate biopsy and the presence of prostate cancer, as well as the Gleason score, was retrospectively collected.

### Magnetic Resonance Imaging

#### Technical Protocol

Every patient underwent multiparametric MRI (mpMRI) of the prostate. The MRI equipment and imaging protocols were made as described in the Prostate Imaging–Reporting and Data System (PI-RADS) version 2.1. MRI was performed on a 1.5-T scanner (MAGNETOM Aera^®^; Siemens Healthcare, Erlangen, Germany), using a dedicated 16-channel phased-array body coil (Siemens Healthcare). Multiplanar (axial, coronal, and sagittal) T2-weighted (T2W) turbo spin-echo (*Z* 263 mm; *X* 350 mm; *Y* 350 mm; voxel size 0.6 × 0.6 × 3.5 mm) and diffusion-weighted MRI (*Z* 193 mm; *X* 200 mm; *Y* 77 mm; voxel size 1.8 × 1.8 × 3.5 mm) were performed with a single-shot echoplanar imaging sequence (*b*-value 50; 800 and 1,500 s/mm^2^). The image software automatically calculated apparent diffusion coefficient (ADC) maps (*Z* 193 mm; *X* 200 mm; *Y* 77 mm; voxel size 1.8 × 1.8 × 3.5 mm). Dynamic contrast-enhanced (DCE) MRI was conducted using a 3D axial gradient echo sequence. Acquisitions were obtained before and after the administration of gadolinium-based contrast medium (gadobutrol Gadovist^®^ 1.0; Bayer Schering Pharma AG, Berlin, Germany) using a dose of 0.1 mmol kg at 1 ml s^−1^, using an automated injector (Ulrich Medical, Ulm, Germany). After the dynamic series, image subtraction of the contrast-enhanced images from the images before the administration of the contrast agent was performed.

#### Feature Extraction

**Figure 1:**
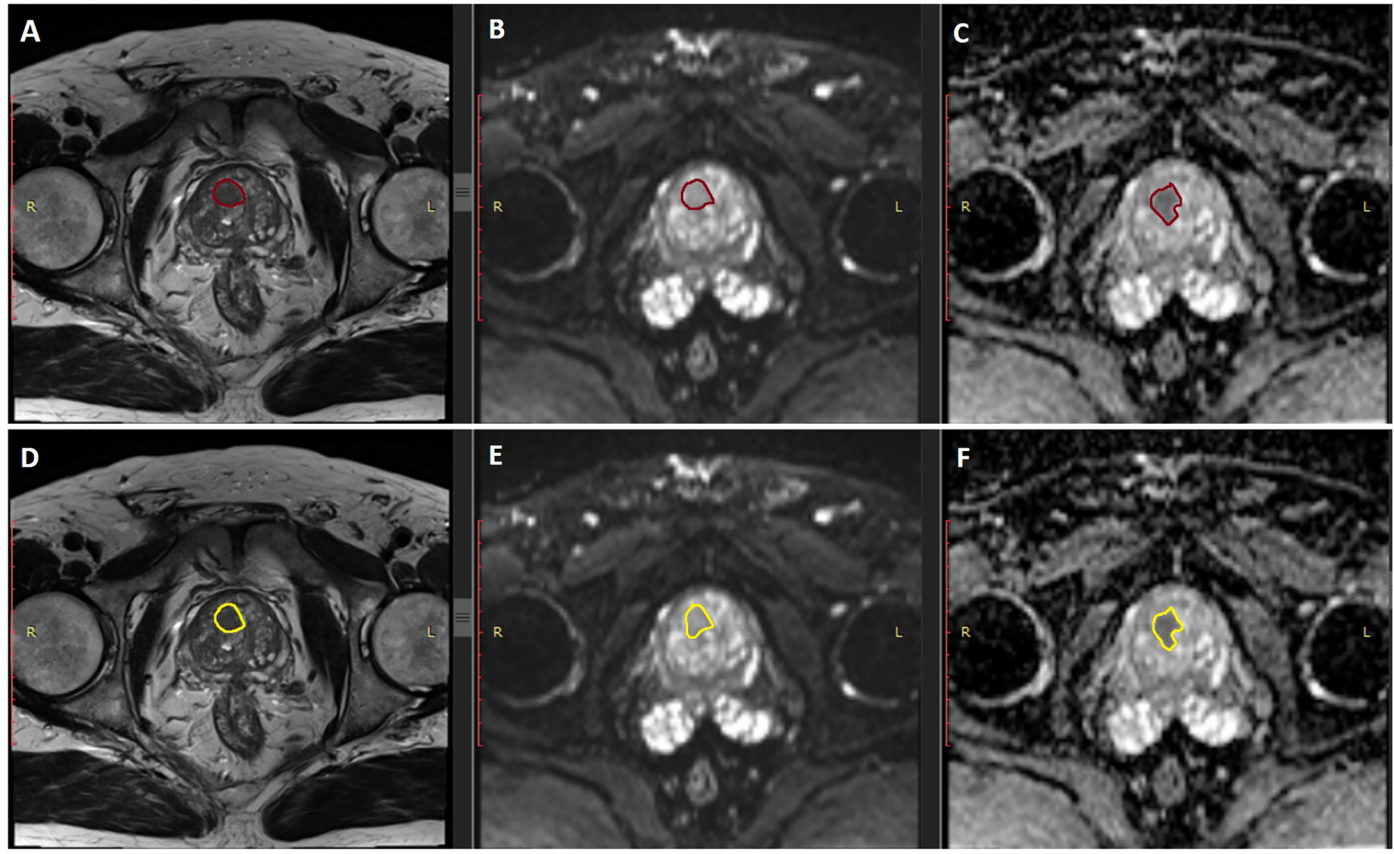
Examples of the two segmentation of the two operators. Panels **(A–C)** represent the examples of segmentation (in burgundy) of the first operator, in the T2, DWI. and ADC MRI sequences, respectively. Panels **(D–F)** represent the examples of segmentation (in yellow) of the second operator, in the same sequences, respectively.

The identification of an MRI suspicious target lesion was done by two radiologists with great experience in prostate cancer (>10 years) (see **Figure 1**).

After the identification, the regions of interest (ROI) were contoured by the same specialists blinded to each other on all the slides where the target lesion was visible. Both the segmentation of the ROI and the extraction of texture features were performed with the freeware software LIFEx^©^ (16). This commercial software is able to extract several features coming from the gray-level histogram, shape, and four matrices of higher order statistics (GLCM, NGLDM, GLRLM, GLZLM) (see **Table 1**).

**Table 1:** Texture analysis parameters calculated with the LIFEx software and the corresponding description.

Type of radiomics feature	Radiomics feature name	Description
Co-occurrence matrix (GLCM): takes into account the arrangements of pairs of voxels to extract textural indices	Homogeneity	Homogeneity of gray-level voxel pairs
	Energy	Uniformity of gray-level voxel pairs
	Correlation	Linear dependency of gray levels in GLCM
	Contrast	Local variations in the GLCM
	Entropy	Randomness of gray-level voxel pairs
	Dissimilarity	Variation of gray-level voxel pairs
Gray-level run length matrix (GLRLM): gives the size of homogeneous runs for each gray level	SRE (short-run emphasis)	Distribution of the short homogeneous runs in an image
	LRE (long-run emphasis)	Distribution of the long homogeneous runs in an image
	LGRE (low gray-level run emphasis)	Distribution of the low gray-level runs
	HGRE (high gray-level run emphasis)	Distribution of the high gray-level runs
	SRLGE (short-run low gray-level emphasis)	Distribution of the short homogeneous runs with low gray levels
	SRHGE (short-run high gray-level emphasis)	Distribution of the short homogeneous runs with high gray levels
	LRLGE (long-run low gray-level emphasis)	Distribution of the long homogeneous runs with low gray levels
	LRHGE (long-run high gray-level emphasis)	Distribution of the long homogeneous runs with high gray levels
	GLNUr (gray-level non-uniformity for run)	Non-uniformity of the gray levels of the homogeneous runs
	RLNU (run-length non-uniformity)	Length of the homogeneous runs
	RP (run percentage)	Homogeneity of the homogeneous runs
Neighborhood gray-level different matrix (NGLDM): corresponds to the difference of gray level between one voxel and its 26 neighborhoods in three dimensions	Coarseness	Level of spatial rate of change in intensity
	Contrast	Intensity difference between neighboring regions
	Busyness	Spatial frequency of changes in intensity
Gray-level zone length matrix (GLZLM): provides information on the size of homogeneous zones for each gray level in three dimensions	SZE (short-zone emphasis)	Distribution of the short homogeneous zones in an image
	LZE (long-zone emphasis)	Distribution of the long homogeneous zones in an image
	LGZE (low gray-level zone emphasis)	Distribution of the low gray-level zones
	HGZE (high gray-level zone emphasis)	Distribution of the high gray-level zones
	SZLGE (short-zone low gray-level emphasis)	Distribution of the short homogeneous zones with low gray levels
	SZHGE (short-zone high gray-level emphasis)	Distribution of the short homogeneous zones with high gray levels
	LZLGE (long-zone low gray-level emphasis)	Distribution of the long homogeneous zones with low gray levels
	LZHGE (long-zone high gray-level emphasis)	Distribution of the long homogeneous zones with high gray levels
	GLNUz (gray-level non-uniformity for zone)	Non-uniformity of the gray levels of the homogeneous zones
	RLNU (zone length non-uniformity)	Length of the homogeneous runs
	ZP (zone percentage)	Homogeneity of the homogeneous zones
Indices from sphericity	Sphericity	Measures how spherical a volume of interest is
	Volume (ml or vx)	Measures the volume in voxels or milliliter
	Surface	Measures the surface of the volume of interest
	Compacity	Measures the degree to which the volume of interest is compact
Indices from histogram: provides information derived from global histogram analysis	Skewness	Measures the asymmetry of the gray-level distribution in the histogram
	Kurtosis	Measures whether the gray-level distribution is peaked or flat relative to a normal distribution
	Min	Measures the minimal value of Hounsfield unit
	Max	Measures the maximal value of Hounsfield unit
	Mean	Measures the mean value of Hounsfield unit
	Std	Measures the standard deviation of the distribution of Hounsfield unit histogram

### Endpoints and Statistical Analysis

Our data consist of the repetition of measurements of texture features for each target lesion identified on MRI imaging. As we want to test the reproducibility and the repeatability of texture features, with different operators, we decided to use intraclass coefficient correlation (ICC) as it is recognized as a method that is independent of the actual scale of measurement and of the size of the error that is considered acceptable (17).

We scored ICC values less than 0.5 as poor reliability, values between 0.5 and 0.75 as moderate reliability, values between 0.75 and 0.9 as good reliability, and values greater than 0.90 as excellent reliability (18–21). ICC estimates and their 95% confidence intervals were based on a mean rating, absolute agreement, two-way mixed-effects model.

The distributions of ICC scores across the different classes of texture features and across the different sequences of mpMRI (multiparametric MRI) were compared with Friedman’s two-way analysis of variance by ranks and with the Wilcoxon signed-rank test. We considered as statistically significant a *p*-value <0.05. All the statistical analysis was calculated using SPSS statistical package version 23 (SPSS Inc., Chicago, IL, USA).

## Results

### Population

Forty-four consecutive patients were included in the present analysis. In 26 patients (59.1%), the prostate biopsy confirmed the presence of prostate cancer, which was scored as Gleason 6 in 6 patients (13.6%), Gleason 3 + 4 in 8 patients (18.2%), and Gleason 4 + 3 in 12 patients (27.3%).

### Reliability of Texture Features

The reliability of texture parameters across different MRI acquisition has shown poor reliability among the two operators in a significant percentage of patients (see **Table 2**).

**Table 2:** Intraclass coefficient correlation (ICC) of the different texture features in the different mrMRI sequences.

Parameter	T2	ADC	DWI 50	DWI 400	DWI 1,500
HIST_min	0.573	0.658	0.346	0.593	0.113
HIST_mean	0.723	0.637	0.317	0.807	0.127
HIST_std	0.644	0.440	0.070	0.596	0.151
HIST_max	0.749	0.587	0.164	0.675	0.204
HIST_Skewness	0.170	0.499	0.395	0.299	−0.273
HIST_Kurtosis	0.302	0.542	0.299	–0.131	0.397
SHAPE_Volume.ml	0.328	0.106	0.118	0.308	0.87
SHAPE_Volume.vx	0.365	0.313	0.196	0.192	0.111
SHAPE_Sphericity	0.025	0.022	0.067	0.155	−0.310
SHAPE_Surface	0.201	0.736	−1.282	0.524	0.004
SHAPE_Compacity	0.076	0.611	−1.480	0.700	−0.114
GLCM_Homogeneity	0.559	0.920	0.713	0.710	0.841
GLCM_Energy	−0.860	0.187	−0.238	0.060	−1.554
GLCM_Contrast	0.601	0.868	0.395	0.684	0.066
GLCM_Correlation	0.286	0.910	0.088	0.728	0.620
GLCM_Entropy_log10	−1.736	0.790	−3.527	0.218	−1.478
GLCM_Entropy_log2	−1.736	0.790	−3.527	0.218	−1.478
GLCM_Dissimilarity	0.630	0.926	0.620	0.741	0.482
GLRLM_SRE	0.378	0.295	0.535	0.717	0.626
GLRLM_LRE	0.374	0.393	0.608	0.571	0.391
GLRLM_LGRE	−0.209	−0.245	−0.100	0.076	−0.320
GLRLM_HGRE	−0.058	0.801	−1.219	0.189	−2.259
GLRLM_SRLGE	−0.201	−0.228	−0.098	0.088	−0.255
GLRLM_SRHGE	−0.065	0.807	−0.784	0.143	−1.719
GLRLM_LRLGE	−0.247	−0.322	−0.115	0.041	−0.492
GLRLM_LRHGE	−0.025	0.771	−0.806	0.594	−5.126
GLRLM_GLNU	0.546	0.347	0.608	0.496	0.015
GLRLM_RLNU	0.310	0.383	0.041	0.102	0.127
GLRLM_RP	0.391	0.379	0.562	0.678	0.639
NGLDM_Coarseness	0.125	0.528	0.515	0.795	0.113
NGLDM_Contrast	0.241	0.101	−0.023	0.170	−0.892
NGLDM_Busyness	0.560	0.137	0.272	0.702	−0.057
GLZLM_SZE	0.513	0.789	0.427	0.863	0.433
GLZLM_LZE	0.257	0.433	0.410	0.952	0.000
GLZLM_LGZE	−0.246	−0.120	−0.141	0.101	−0.276
GLZLM_HGZE	−0.146	0.827	−0.836	−0.185	−0.816
GLZLM_SZLGE	−0.165	0.039	−0.110	0.214	0.345
GLZLM_SZHGE	0.011	0.877	0.535	−0.111	−0.152
GLZLM_LZLGE	0.124	−4.067	0.417	−0.104	−0.011
GLZLM_LZHGE	0.355	0.179	0.106	0.972	0.000
GLZLM_GLNU	0.332	0.481	0.285	0.470	0.284
GLZLM_ZLNU	0.003	0.637	−0.443	0.106	−0.241
GLZLM_ZP	0.669	0.811	0.477	0.916	0.656
Poor reliability (ICC < 0.5)	32 (74%)	22 (51%)	35 (82%)	23 (54%)	37 (86%)
Moderate reliability (ICC 0.5–0.75)	11 (26%)	7 (16%)	8 (18%)	14 (32%)	4 (9%)
Good reliability (ICC 0.75–0.9)	0	11 (26%)	0	3 (7%)	2 (5%)
Excellent reliability (ICC > 0.9)	0	3 (7%)	0	3 (7%)	0

In bold the classification of the texture features.

**Figure 2:**
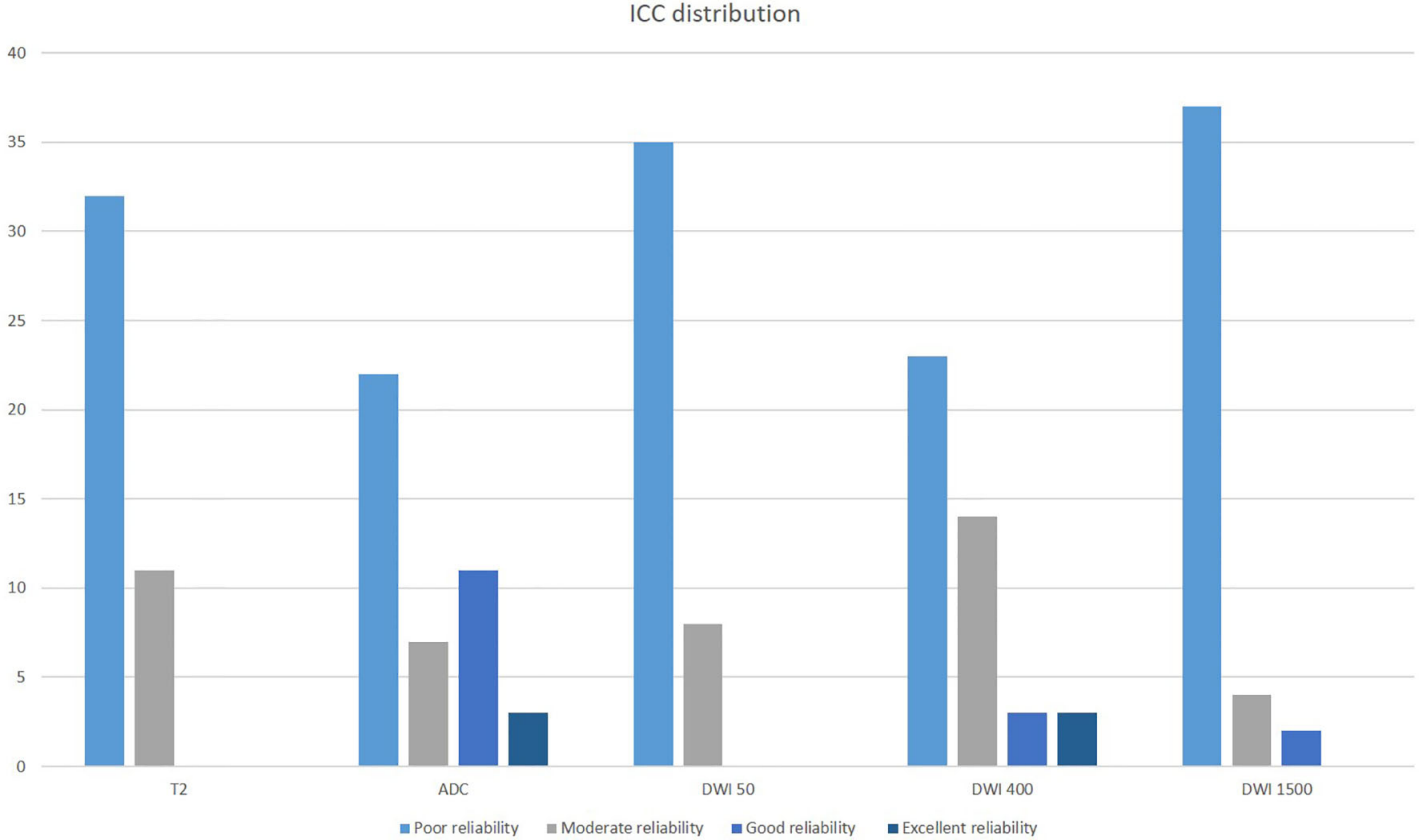
Intraclass coefficient correlation (ICC) distribution of texture features across the different mpMRI imaging acquisitions.

Specifically, ICC was scored as poor in 74% of T2, in 22% of ADC, in 82% of DWI50, in 54% of DWI400, and in 86% of DWI1,500 (see **Figure 2**). The ADC sequences showed scores with better reliability, with 33% of the texture parameters scored as good and excellent ICC. The median values of ICC were, respectively, 0.25 ± 0.53 (T2), 0.49 ± 0.77 (ADC), 0.11 ± 0.92 (DWI50), 0.47 ± 0.33 (DWI400), and 0.12 ± 0.93 (DWI1,500).

### Correlation of ICC With MRI Sequences and Radiomics Analysis Subsections

**Figure 3:**
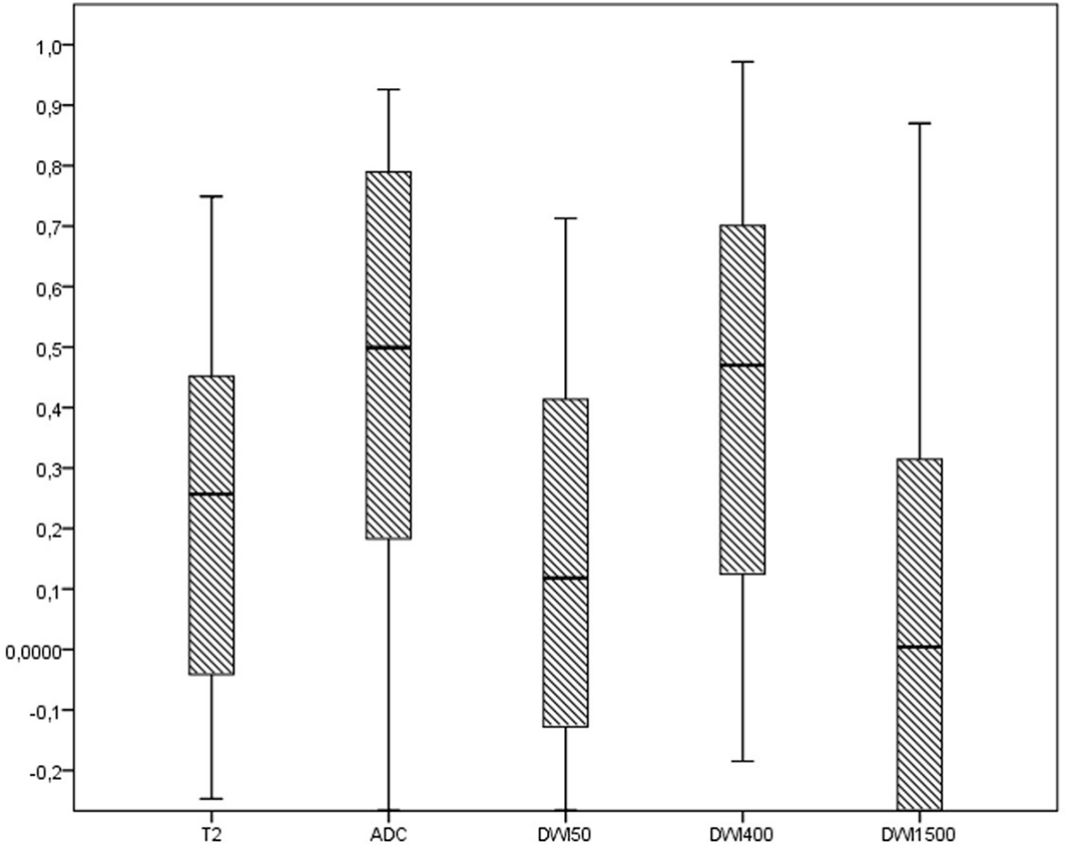
The distribution of ICC of radiomics features across the different MRI sequences.

The distribution of ICC across the different subsections of radiomics analysis in the different MRI sequences was significantly different in histogram (*p*: 0.046), GLCM (*p*: 0.001), GLRLM (*p*: 0.014), and GLZLM features (*p*: 0.004) and was the same in NGLDM (*p*: 0.066) and shape features (*p*: 0.326) (see **Figure 3**).

**Figure 4:**
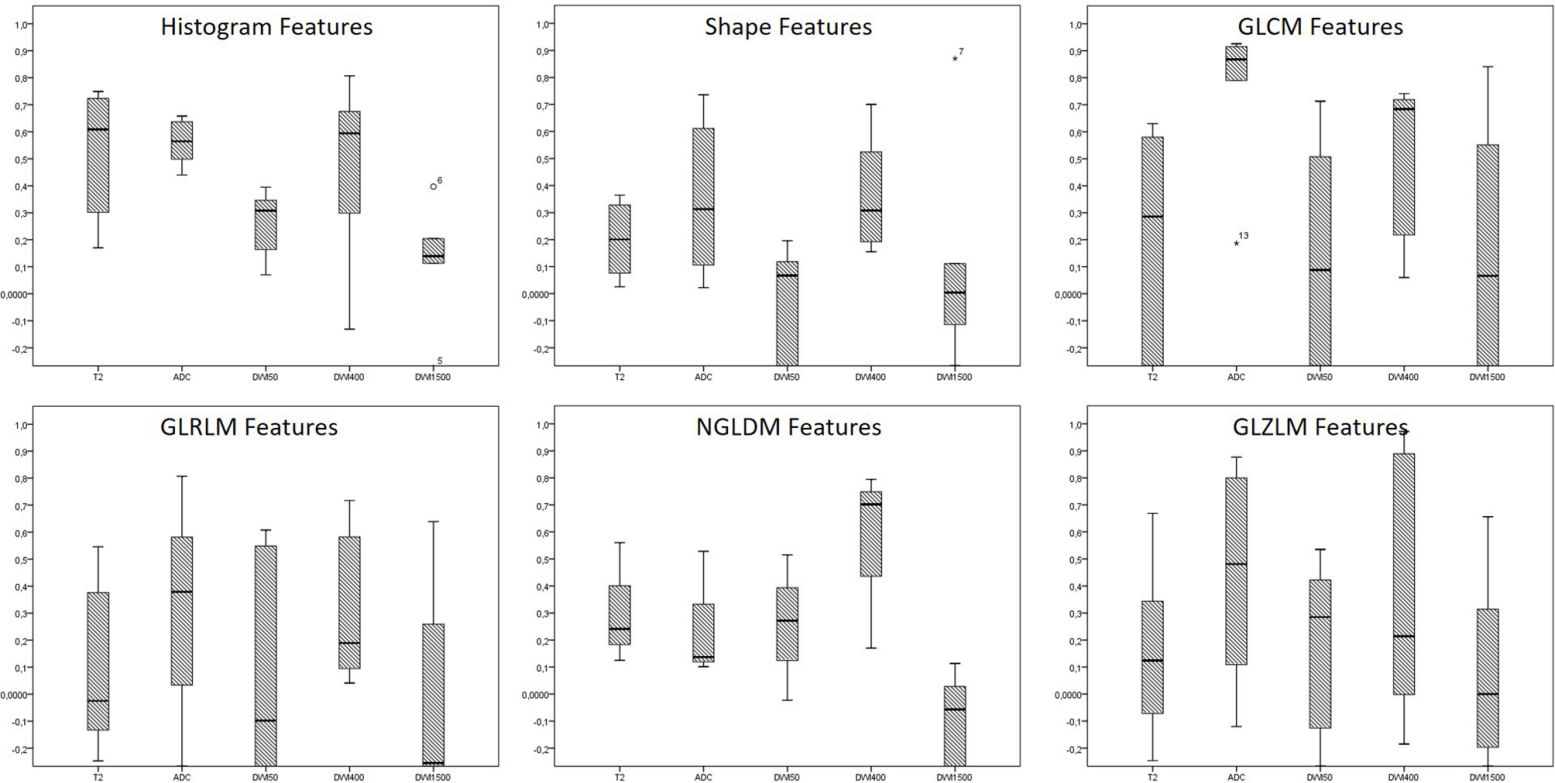
The distribution of ICC across the different subclasses of radiomics features in the different MRI sequences. * are the outliers.

Considering the ADC and the DWI400 sequences as the most reliable sequences, there were significant differences in the distribution of ICC in GLCM features (*p*: 0.018), with no differences in the other subsections of shape features (*p*: 0.893), GLRLM (*p*: 0.594), NGLDM (*p*: 0.109), and GLZLM (*p*: 0.594) (see **Figure 4**).

## Discussion

Prostate cancer involves tumors with different biological patterns and characteristics and, consequently, different prognoses. The therapeutic approach is a field of fervent debate especially in localized stages, with different strategies being adopted (22, 23).

A deeper knowledge of the specific biological pattern is needed to provide the correct treatment in each PCa patient. Biological and molecular analysis had already been correlated with patterns of disease, and nowadays, radiomics represents an increasingly interesting frontier in the oncology field (10, 24). However, there are still many limitations for the analysis of quantitative parameters through dedicated software to enter the routine diagnostic pipeline, relying on the reproducibility of collected data. MpMRI is routinely used as part of the diagnostic workup and staging algorithm in PCa (25). In fact, recent studies suggest that mpMRI reduces the number of unnecessary prostate biopsies in patients with PI-RADS score of 3 or more. T2, DWI, and ADC are the most commonly employed sequences assessing PI-RADS score. ADC could heavily support clinical workflow in decision-making for patients with PI-RADS score <3 who are considered at risk for PCa (26, 27).

Thus, the implementation and validation of radiomic features is seemingly feasible and convenient, to gather more information with limited additional costs as well as avoid invasive diagnostic procedures (e.g., transrectal gland biopsy).

In radiomics, it is of paramount importance to understand that most of the features were originally developed for non-medical imaging and for planar images. In the context of clinical investigation, the final aim of radiomics is to use texture features as surrogate biomarkers of different clinical endpoints. Thus, surrogate biomarkers must be correlated to the endpoint, and at the same time, their mensuration must be accurate and robust (28, 29).

Every single process in the workflow of radiomics (image acquisition and reconstruction, segmentation, feature extraction, data analysis, model building, and validation) has its own challenges, and in particular, the challenges relevant to robustness of radiomics features has been analyzed by many researchers in recent years (30, 31).

Our study focused on reliability validation of specific radiomic parameters extracted from mpMRI in a diagnostic setting, while recently published reports in the application of radiomics at a therapeutic level investigated radiomics feature reliability carrying out a thorough assessment of repeatability and reproducibility of MRI radiomics features in MRI-guided radiotherapy (MRgRT) in PCa (32). Similar to our results, in this study, the differences originating from MRI acquisition were notably described as the most impactful on reliability (33), and only a few features showed good to excellent repeatability and reproducibility coefficients, although such identification is still insufficient for a reliable radiomics study (32). The observed reliability is poor also in other studies, in accordance with our results (34, 35).

Detection of PCa lesions using radiomics feature extracted from mpMRI (36) images and an objective increase in sensitivity and specificity in detecting PCa (37) with computer-aided diagnosis tools are already possible although non-negligible uncertainties may occur. Moreover, in Gleason and PI-RADS prediction, texture-based features, geometric parameters, and contrast and homogeneity GLCM features in different studies have shown radiomics-augmented capability to predict GS. The future uses of radiomics could involve aftertreatment evaluation of biochemical recurrence risk, to better stratify patients after radical prostatectomy, therefore helping the clinician to adapt postoperative management.

Despite its potential, the low reproducibility of radiomics approaches represents a significant hurdle to enter the routine clinical workup of PCa.

Homogeneous protocols based on radiomics are yet to be developed in both diagnostic and therapeutic settings. The poor reliability of datasets does not allow comparisons between cohorts of different patients.

The causes of low reliability are several and complex to analyze. A fundamental role is played by the experience of the operator, but given the relatively recent development of radiomics, it is rare to identify operators with significant and consistent experience.

Furthermore, the diagnostic assessment of PCa through mpMRI has always been challenging far earlier than the radiomics approach, due to the difficulties and heterogeneity in prostate cancer segmentation and PI-RADS score assignment (38–40). To date, no guidelines are available for contouring prostate cancer inside the gland, as visible on mpMRI, and the analysis of the contourings of the multicenter phase III FLAME trial showed significant different interpretations in tumor contouring between institutes (40).

In this regard, sharing images, radiomics feature, and contouring guidelines and the implications derived from them represent an unmet need that must be solved in the near future. Further studies on genomics feature are needed to face the poor reliability issue, empowering the role of radiomics applications for PCa.

## Limitations

We recognize many limitations in our work. Firstly, we performed a retrospective analysis of a single cohort of homogeneous patients with suspicious prostate cancer. At the same time, the number of analyzed patients is low.

## Conclusions

In our study, we demonstrated that the reliability of MRI features in prostate cancer is extremely low. These findings reinforce the pivotal importance of preclinical studies before applying radiomics in clinical practice.

## Data Availability Statement

The raw data supporting the conclusions of this article will be made available by the authors, without undue reservation.

## Ethics Statement

Ethical review and approval was not required for the study on human participants in accordance with the local legislation and institutional requirements. The patients/participants provided their written informed consent to participate in this study.

## Author Contributions

VN, AR, FU, and SC conceived and designed the study and wrote the manuscript. VP, AA, LD’A, PR, GR, LG, and MC acquired the clinical data. VN performed the statistical analysis. VN, FU, CV, AR, and SC followed the patients, including planning clinical visits, blood sample collection, and follow-up. LA, SC, and AR revised the paper. All authors read and approved the submitted version of the manuscript.

## Conflict of Interest

The authors declare that the research was conducted in the absence of any commercial or financial relationships that could be construed as a potential conflict of interest.

## Publisher’s Note

All claims expressed in this article are solely those of the authors and do not necessarily represent those of their affiliated organizations, or those of the publisher, the editors and the reviewers. Any product that may be evaluated in this article, or claim that may be made by its manufacturer, is not guaranteed or endorsed by the publisher.
